# Navigating Complexity: A Case Report on a Comprehensive Dental Management Approach to Amelogenesis Imperfecta and Gingival Fibromatosis Syndrome

**DOI:** 10.7759/cureus.53787

**Published:** 2024-02-07

**Authors:** Hitaf Nasrallah, Khetam Berro

**Affiliations:** 1 Department of Pediatric Dentistry, Lebanese University Faculty of Dental Medicine, Beirut, LBN

**Keywords:** multidisciplinary approach, genetic disorder, mixed dentition, gingival hyperplasia, amelogenesis imperfecta, aigfs

## Abstract

This clinical case report details the comprehensive diagnosis and dental management of a seven-year-old female patient diagnosed with the rare genetic disorder, amelogenesis imperfecta and gingival fibromatosis syndrome (AIGFS). The case initially presented as congenital adrenal hyperplasia and amelogenesis imperfecta, but further genetic analysis revealed the involvement of AIGFS due to a mutation in the *FAM20A* gene. Diagnosis, confirmed through whole exome sequencing, clinical assessment, and laboratory tests, necessitated a multidisciplinary approach to address the treatment of such cases. The article underscores the critical importance of diagnosing and managing dental manifestations in pediatric patients with complex genetic conditions, highlighting the difficulties of treating AIGFS in mixed dentition. This case also highlights the indispensable role of pediatric dentists in diagnosing and treating these cases, ultimately improving the quality of life for individuals with AIGFS.

## Introduction

Amelogenesis imperfecta (AI) encompasses a diverse range of genetically and clinically variable inherited dental enamel abnormalities. While it is often characterized as a standalone condition, it can also manifest alongside additional oro-dental and/or systemic characteristics. Examples include the co-occurrence of nephrocalcinosis in cases of enamel renal syndrome (ERS, MIM#204690) and gingival hyperplasia in instances of amelogenesis imperfecta and gingival fibromatosis syndrome (AIGFS, MIM#614253) [1].

Hereditary gingival fibromatosis is a rare disorder characterized by excessive fibrous growth of gingival tissue, which may manifest either independently or as a component of a syndrome or chromosomal irregularity [2,3].

AIGFS is an autosomal recessive condition marked by mild gingival fibromatosis and dental anomalies, encompassing hypoplastic AI, intrapulpal calcifications, delayed tooth eruption, hypodontia/oligodontia, pericoronal radiolucencies, and unerupted teeth [4].

Enamel acts as a physical barrier because of the hardness and the highly mineralized nature of this tissue. The significance of the enamel's role is particularly evident in hypomineralized conditions, emphasizing its crucial importance. For instance, among patients with hypomineralized AI, the enamel shows normal thickness, but mineralization is defective. Consequently, the tissue does not play its protective role, resulting in tooth sensitivity [5]. From a biological standpoint, enamel defects become local risk factors for bacterial adhesion and plaque colonization [6,7], particularly when observed among patients with hypomineralized and hypoplastic AI.

To avoid dental caries, gingival inflammation, open bite, or vertical dimension loss, it is advisable to pursue interdisciplinary patient care. Specifically, successful oral rehabilitation relies on essential conservative, prosthetic, and orthodontic treatments. The choice of treatment options varies depending on factors such as the patient’s age, socioeconomic conditions, and the extent of malformation [8]. Although stainless steel crowns, strip crowns, and compomer restorations are frequently used in primary dentition, managing dentition becomes a challenge during the growth phase in adolescents, especially in mixed and permanent dentition [9]. The management of individuals affected by AI is typically categorized into three distinct stages, as outlined in the literature [10]. The initial stage, referred to as the temporary phase, is implemented during the primary and mixed dentition. Following this, the transitional phase commences with the eruption of all permanent teeth and extends into adulthood. Finally, the permanent phase of management is initiated during adulthood.

Nevertheless, despite the fact that AI may often be diagnosed clinically, many of the associated diseases may remain masked and present a challenge in detection. Consequently, we present a case of a seven-year-old female patient diagnosed with AIGFS, with the added complexity of managing mixed dentition. This paper discusses the specific dental treatments applied, highlighting the careful approach taken to address the unique challenges presented by AIGFS.

## Case presentation

**Figure 1:**
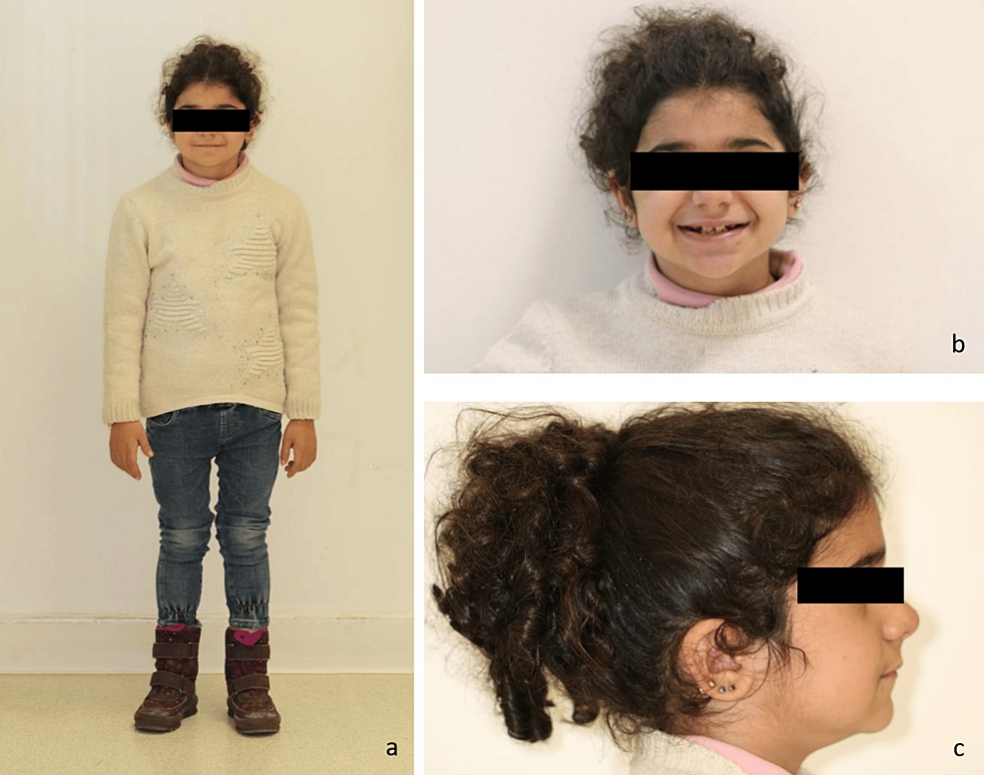
The patient's extraoral clinical examination, highlighting facial features associated with amelogenesis imperfecta and gingival fibromatosis syndrome (a) Patient standing showing short stature and wasted appearance. (b) Frontal and (c) lateral extraoral photographs showing a straight facial profile, low hairline, thick eyebrows, and facial hair.

A seven-year-old female patient presented with complaints of teeth sensitivity and aesthetic concerns related to her dentition. The patient expressed dissatisfaction with the appearance of her teeth, describing them as having an unattractive yellowish hue. The patient's medical history revealed a diagnosis of congenital adrenal hyperplasia (CAH), for which she was receiving 10 mg of corticosteroids daily. She was the only child of consanguineous parents, having no reported history of CAH, AI, or gingival fibromatosis in the family. Extraoral clinical examination showed short stature, a wasted appearance (Figure 1a) balanced facial proportions, a straight profile, low hairline, thick eyebrows, and facial hair (Figures 1b, 1c).

**Figure 2:**
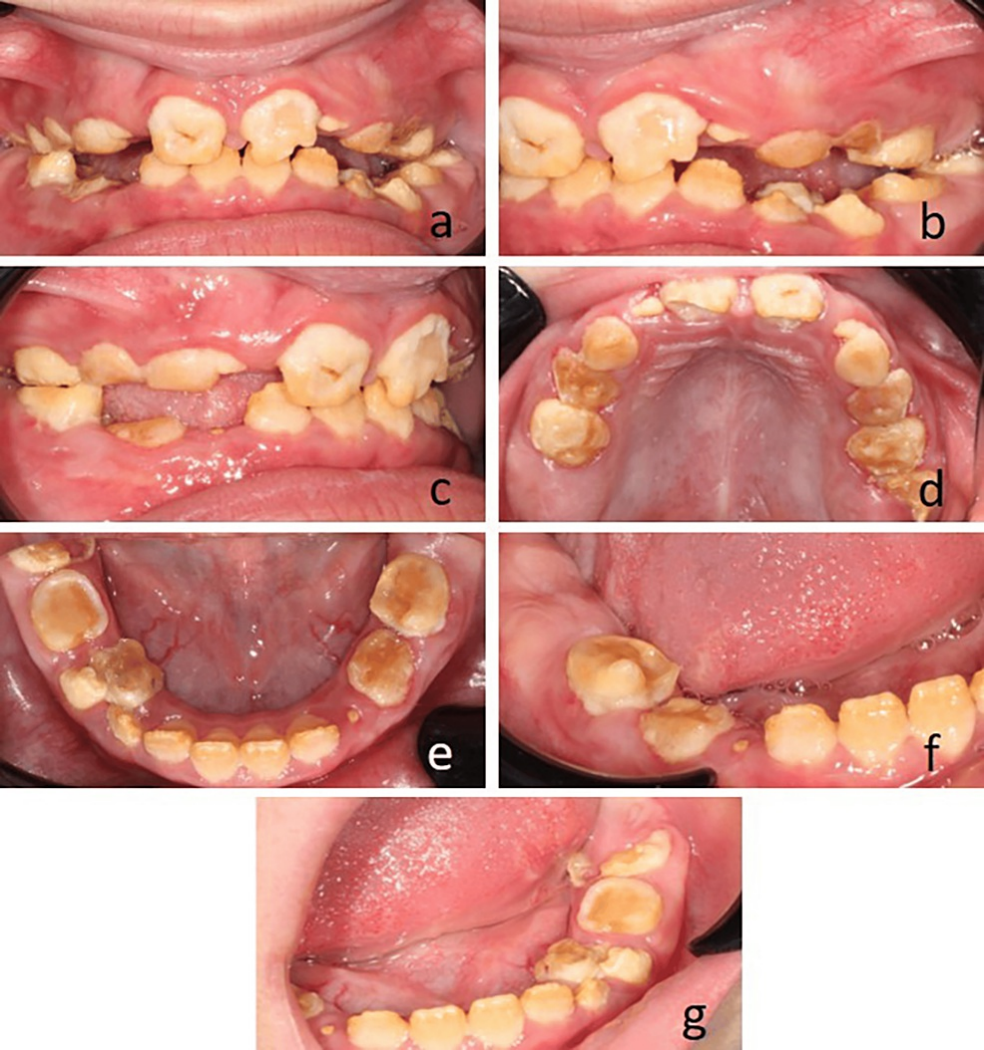
Intraoral examination highlighting enamel defects observed in the patient diagnosed with amelogenesis imperfecta and gingival fibromatosis syndrome (a) Frontal view showing hypoplastic chipped anterior teeth. (b) Lateral left intraoral photo showing class I. (c) Left lateral view showing class I, with both overjet and overbite. (d) Upper arch occlusal view showing gingival coverage of 16 and partial eruption of 26. (e) Lower arcade occlusal view showing flat cusps. (f) Lower right lateral view showing full gingival coverage of 46. (g) Lower left lateral view showing partial coverage of 36.

Upon intraoral inspection, a bilateral dental class I occlusal relationship was identified, with both overjet and overbite falling within the normal range (Figures 2b, 2c). Additionally, it was observed that the patient exhibited a mixed dentition characterized by delayed development relative to her chronological age. The intraoral examination also revealed significant wear of the occlusal surfaces (Figures 2e-2g), attributed to the defective enamel surface resulting in decreased vertical dimension. The clinical crowns appeared shortened due to issues related to AI. Enamel presented as chalky, rough, and scratchy in texture, indicative of compromised structural integrity, which confirmed the diagnosis of AI. In further detailing the intraoral examination, teeth 16 and 46 were entirely covered by gingival tissue (Figures 2d, 2f). Additionally, teeth 26 and 36 showed partial eruption (Figures 2d, 2g). Tooth 54 presented with eroded enamel surfaces. Additionally, there were widespread fibrotic gingival enlargements accompanied by secondary inflammation, affecting nearly all teeth. A provisional diagnosis of hypoplastic AI was proposed along with a differential diagnosis of environmental enamel hypoplasia, dentinogenesis imperfecta, and dentin dysplasia. For the gingiva, a provisional diagnosis of plaque-induced hyperplasia with a differential diagnosis of hereditary familial fibromatosis was made.

**Figure 3:**
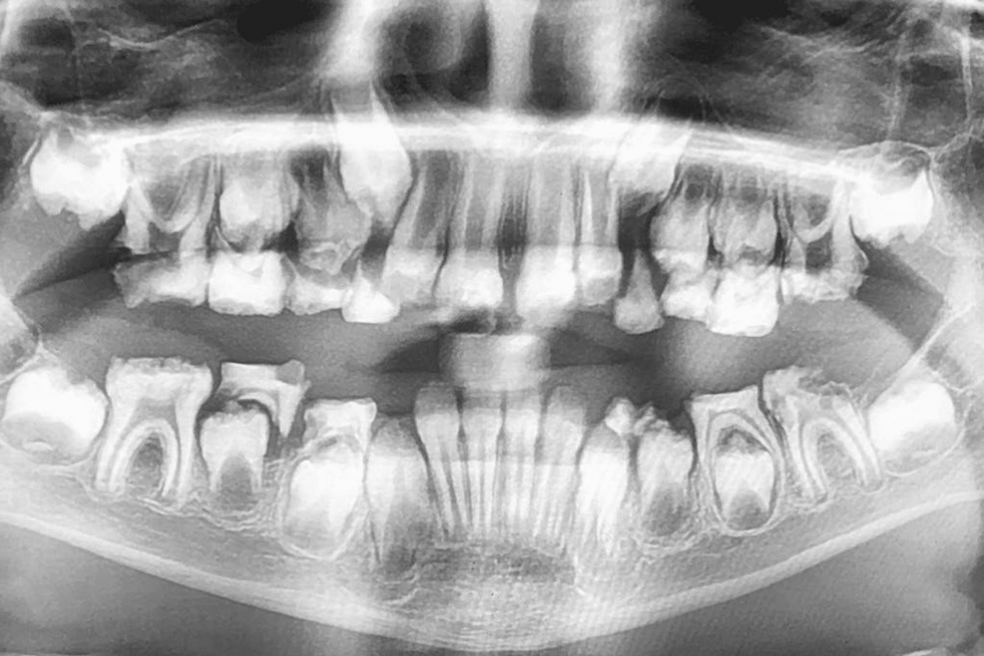
Advanced shedding of primary teeth, thin enamel layers, and teeth with flat cusps.

Radiographic examination (Figure 3) by panoramic X-ray showed advanced shedding of primary teeth, appearance of thin or absent enamel layers on the radiograph, abnormal crown shapes, increased attrition or wear patterns on the tooth surfaces caused by hypoplastic enamel, and, in some instance, enlarged pulp chamber (taurodontism).

Whole exome sequencing was performed, which revealed that the patient is homozygous for a known pathogenic variant in the *CYP21A2* gene: CAH is caused by homozygous or compound heterozygous mutation in the *CYP21A2 *gene. It also revealed that the patient is homozygous for a known likely pathogenic variant in the *FAM20A* gene: AI type IG, also known as enamel-renal syndrome, is caused by homozygous or compound heterozygous mutation in the *FAM20A* gene.

Given the extensive nature of the required care, the patient underwent a thorough rehabilitation conducted over five sessions, during which a conscious sedation method with nitrous oxide/oxygen was administered (30% NO_2_ and 70% O_2_) with a scented nasal mask (DynoMite, Matrx, Porter Instrument Company, Inc, Hatfield, PA) and nitrous oxide machine (Porter MXR Flowmeter, Porter Instrument Company, Inc) to address her age-related and anxiety-related consideration. The treatment approach involved a combination of dental and periodontal interventions to address the enamel defects and gingival overgrowth associated with the condition.

**Figure 4:**
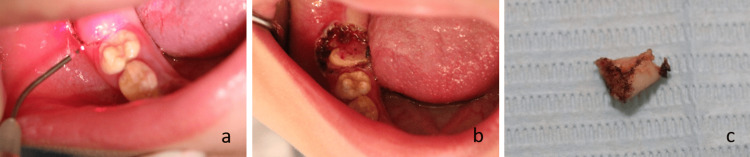
Gingivectomy. (a) Application of laser therapy using 980-nm diode laser. (b) Exposed tooth 46 showing flat cusp. (c) Thick gingival tissue.

Since she had mixed dentition, the treatment goals were to maintain tooth vitality, decrease tooth sensitivity, fix vertical dimension, manage gingival overgrowth, expose teeth, and improve aesthetics. Oral hygiene instructions, prophylaxis, and fluoride gel application were performed, followed by vertical dimension augmentation after exposing the teeth with laser gingivectomy procedures. One of the main advantages of increasing the Vertical dimension of occlusion in such cases is to provide a space for restorative material as minimum occlusal reduction is needed. During the laser treatment, both the operator and the patient wore protective eyewear. Laser irradiation was applied using a 980-nm diode laser (Medency Primo 10 W Diode Laser, Vicenza, Italy) coupled with a 200-µm optical fiber (spot size of 0.02 cm in diameter and an area of 0.000314 cm^2^) (Figure 4a). The settings were as follows: output power of 6.50 W and energy of 30 J, pulsed mode with 25 ms time ON, and 50 ms time OFF. Laser gingivectomy was performed around teeth 16, 26, 36, and 46, exposing teeth with abnormal morphology flat cusps and brittle enamel (Figure 4b), as well as gingival tissue that was relatively hard (Figure 4c).

**Figure 5:**
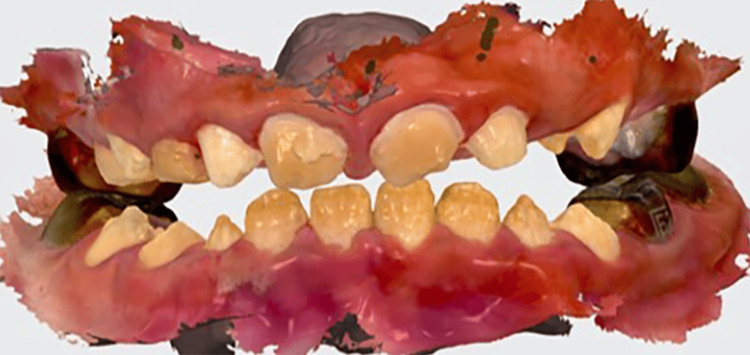
Digital impression taken by TRIOS 3: upper and lower arches.

**Figure 6:**
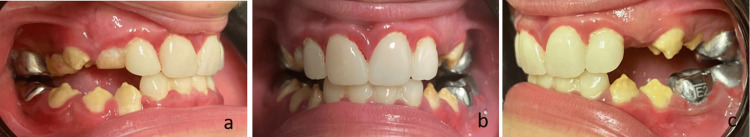
Outcome of the rehabilitation process, showcasing the improved dental condition and aesthetic restoration achieved through comprehensive treatment.

An incisional biopsy from two areas, one from the lower right partially erupted canine and the other from the gingiva of the lower left molar, revealed dystrophic calcifications associated with AIGFS [1]. In the same session after gingivectomy, a therapeutic approach was taken with mineral trioxide aggregate (MTA) pulpotomy (Biodentine™, Septodont, Saint-Maur-des-Fossés, France), and stainless steel crowns (3M, Neuss, Germany) were applied in order to prevent gingival overgrowth over the next session and control bleeding. These treatments were performed for permanent first molars 16, 26, 36, and 46; following the tangential preparation, contouring, and crimping, well-fitting metal crowns are "snapped" in place and then cemented with glass ionomer cement (Ketac™ Cem Aplicap™, 3M/ESPE, St Paul, MN, USA). The patient was put on chlorhexidine rinse twice per day for TWO weeks. Additionally, pulpotomy with reinforced zinc oxide eugenol (intermediate restorative material) was then conducted on teeth 65 and 75, followed by the placement of stainless steel crowns. After fixing the vertical dimension, digital impression for the upper incisor teeth 11, 12, 21, and 22 and the lower incisor teeth 31, 32, 41, and 42 was made using an intraoral scanner TRIOS 3® (3Shape, Copenhagen, Denmark) (Figure 5). The choice of digital impression is more comfortable and preferable for children [11]. Laboratory-fabricated full-coverage composite crowns were chosen because of the noticed chipping of her teeth with time. In the last session, the crowns were cemented using Panavia (Panavia SA, Kuraray-Noritake, Kurashiki, Japan) (Figures 6a-6c).

## Discussion

This report outlines the case of a pediatric patient diagnosed with AIGFS through whole exome sequencing, clinical examination, and laboratory assessments.

In 2008, Martelli-Junior et al. described the cases of four patients (from a consanguineous family) with gingival hyperplasia and dental abnormalities including generalized thin hypoplastic AI, with an autosomal recessive inheritance [12]. Thin generalized hypoplastic AI, intrapulpal calcifications, delayed tooth eruption, and failure of tooth development were described by O’Sullivan et al. in 2011 [13]. Cho et al. in 2012 reported the case of a large consanguineous family affected by AI with gingival hyperplasia, as well as generalized hypoplastic enamel, failed eruption of permanent teeth with dilaceration of the root, severe generalized gingival hyperplasia, and agenesis of the left mandibular second premolar [14]. In 2015, the first reported case in Morocco of AIGFS by Cherkaoui et al. showed hypoplastic AI affecting primary and permanent dentition, generalized gingival hyperplasia, crowns that were short, yellow-brown, and covered with little or no enamel, and supra-incisive diastema [15]. AIGFS cases were reported by different countries all over the world. To our knowledge, this is the first case report on a Lebanese patient with AIGFS.

This case had, in the first place, diagnostic challenges. Distinguishing between ERS and AIGFS presented significant challenges due to overlapping clinical and radiographic features, particularly hypoplastic AI and gingival abnormalities. The complexity deepened with the coexistence of CAH, further highlighting the importance of genetic testing and multidisciplinary collaboration.

The collaboration of endocrinologists, pediatric nephrologists, genetic counselors, and pediatric dentists played a pivotal role in navigating the diagnostic and treatment complexities. The integration of insights from pediatric dentistry alongside medical evaluations, laboratory analyses, and advanced imaging contributed to a holistic understanding of the patient's condition, emphasizing the need for a unified approach to managing complex cases with diverse clinical manifestations

Rehabilitating a patient with AIGFS poses challenges in addressing both functional and aesthetic aspects. The presence of mixed dentition presented an initial treatment challenge, which was further complicated by gingival overgrowth, obstructing the eruption of permanent teeth. The treatment strategy focused on addressing gingival overgrowth through laser intervention to control bleeding. Simultaneously, within the same session, MTA pulpotomy was performed to manage dental pulp-related issues associated with AI.

Full coverage crowns were chosen instead of composite veneers because enamel defects involve proximal surfaces, and decreased bonding is expected; in addition, these crowns offer reasonable esthetics and retention due to their full coverage and the use of cement.

## Conclusions

This case study emphasizes the critical role of pediatric dentists in the holistic care of individuals with complex genetic conditions, advocating for an integrated approach that extends beyond traditional dental interventions. The successful management of this challenging case not only improved the patient's oral health and aesthetic concerns but also highlighted the broader implications for enhancing the quality of life for individuals affected by rare genetic disorders. As advancements in genetic research and interdisciplinary collaboration continue, such cases provide valuable insights into refining treatment strategies and optimizing outcomes for patients with unique and complex dental conditions.
